# Draft Genome Sequences of *Spirosoma agri* KCTC 52727 and *Spirosoma terrae* KCTC 52035

**DOI:** 10.1128/MRA.00317-20

**Published:** 2020-06-04

**Authors:** Julian Rojas, Binoy Ambika Manirajan, Stefan Ratering, Christian Suarez, Sylvia Schnell

**Affiliations:** a Institute of Applied Microbiology, Justus-Liebig-University, Giessen, Germany; b School of Biosciences, Mahatma Gandhi University, Kerala, India; University of Maryland School of Medicine

## Abstract

*Spirosoma agri* S7-3-3 (KCTC 52727) and *Spirosoma terrae* 15J9-4 (KCTC 52035) are type strains isolated from an apple orchard and beach soil in South Korea, respectively; their draft genome sequences were assembled and annotated. The draft genome sequences of S7-3-3^T^ (7,239,915 bp; G+C content, 50.6%) and 15J9-4^T^ (7,551,610 bp; G+C content, 47.3%) are reported.

## ANNOUNCEMENT

*Spirosoma* is the largest genus in the family *Cytophaga*, class *Bacteroidetes*. Recently described species have been isolated from several environmental habitats, such as air, dust, water, and soil (1–4).

Typical characteristics of the group members are the following: diversity of morphology such as rods, coils, and filaments; Gram-negative staining; colonies yellow to orange pigmented; phosphatidylethanolamine as the major polar lipid; MK7 as the major menaquinone; summed feature 3 (C_16:1_ *ω*7c/C_16:1_ *ω*6c) as the major fatty acid; and DNA G+C content range of 47.2 to 57.0 mol% (2, 3, 5–8).

*Spirosoma agri* S7-3-3^T^ was described by Li et al. (3) and *Spirosoma terrae* 15J9-4^T^ by Ten et al. (2), and both were validly published according to the International Code of Nomenclature of Prokaryotes (9). Both strains are Gram-negative, nonmotile, rod-shaped bacteria initially isolated from apple orchard soil in Gyeongsangnam, South Korea, and from soil collected on Jeju Island, South Korea, respectively, using a dilution plating method on R2A agar (Difco). Both grow optimally in R2A medium at 25°C and pH 7.0. According to the 16S rRNA gene similarities, the closest relatives to *S. agri* S7-3-3^T^ were Spirosoma rigui WPCB118^T^ (94.3%) and Spirosoma pulveris JSH5-14^T^ (93.9%) (3), and those for *Spirosoma terrae* 15J9-4^T^ were Spirosoma panaciterrae Gsoil 1519^T^ (94.2%) and Spirosoma luteolum 16F6E^T^ (94.1%) (2).

*S. agri* S7-3-3^T^ (KCTC 52727) and *S. terrae* 15J9-4^T^ (KCTC 52035) were purchased from the Korean Collection for Type Cultures (KCTC). The total genomic DNA for each strain was obtained using the method of Pitcher et al. (10) after growing the colony in R2A liquid medium for 48 h at 25°C. Two paired-end libraries were sequenced using 300-bp paired-end chemistry on a MiSeq v3 sequencer system (Illumina) at LGC Genomics (Germany). The sequencing yielded 1,780,390 raw reads for *Spirosoma agri* S7-3-3^T^ and 1,350,368 for *Spirosoma terrae* 15J9-4^T^, and quality control of the reads was assessed with FastQC (11). The reads were assembled with SPAdes v3.13.1 (12) using k-mer values of 21, 33, and 55. Open reading frames (ORFs), gene annotation, and G+C contents were determined using the NCBI Prokaryotic Genome Annotation Pipeline (PGAP) (13). Genome completeness and contamination were assessed with CheckM v1.0.18 using default parameters (14). Carbohydrate-active enzymes (CAZymes) were annotated with the dbCAN database using model HMMdb v8.0 (E value, <1e^−15^; coverage, >0.35) (15), and secondary metabolite biosynthesis gene clusters were identified using antiSMASH v5.0.0 with default parameters (16). Information about sequencing and annotation results is summarized in Table 1. Based on CheckM, the draft genomes were estimated to be ≥99% complete with <1.2% contamination.

**TABLE 1:** Sequencing and annotation results for *S. agri* S7-3-3^T^ and *S. terrae* 15J9-4^T^

Strain	Assembly size (bp)	No. of contigs	*N*_50_ (bp)	No. of predicted coding sequences	No. of:		G+C content (%)
					tRNAs	rRNAs (5S, 16S, 23S)	
*S. agri* S7-3-3^T^	7,239,915	36	4,167,621	5,826	41	3 (1, 1, 1)	50.6
*S. terrae* 15J9-4^T^	7,551,610	62	365,996	6,170	43	4 (1, 2, 1)	47.3

Genome annotation revealed genes for nitrate reduction in *S. agri* S7-3-3^T^ but not in *S. terrae* 15J9-4^T^; furthermore, both strains have genes for alkaline phosphatase, cellulase, and amylase activity.

The dbCAN analysis described 355 genes for *S. agri* S7-3-3^T^ and 314 genes for *S. terrae* 15J9-4^T^ encoding proteins for carbohydrate binding, carbohydrate esterases, glycoside hydrolases, and glycoside transferases. Additionally, using antiSMASH, gene clusters for the production of ladderane, terpene, polyketide synthase types I and III (T1PKS, T3PKS), and nonribosomal peptide synthetase (NRPS) were annotated. These genomes will contribute to the genomic knowledge of the members of genus *Spirosoma*.

### Data availability.

The genome sequences of these two strains have been deposited in GenBank; the raw data sets can be found under BioProject accession numbers PRJNA590610 for *S. agri* S7-3-3^T^ and PRJNA590616 for *S. terrae* 15J9-4^T^. The assembled sequences for *S. agri* S7-3-3^T^ (BioSample accession number SAMN13335970) can be accessed under accession number ASM1074741v1; the assembly version described in this paper is the first version. For *S. terrae* 15J9-4^T^ (BioSample accession number SAMN13335992), the assembled sequences can be accessed under accession number ASM1043591v1; the assembly version described in this paper is the first version.
